# Trastuzumab induced *in vivo* tissue remodelling associated *in vitro* with inhibition of the active forms of AKT and PTEN and RhoB induction in an ovarian carcinoma model

**DOI:** 10.1038/sj.bjc.6605699

**Published:** 2010-06-01

**Authors:** J-P Delord, S Quideau, P Rochaix, O Caselles, B Couderc, I Hennebelle, F Courbon, P Canal, B C Allal

**Affiliations:** 1 Laboratoire de Pharmacologie Clinique et Expérimentale des Médicaments Anticancéreux (EA 3035) Université Paul Sabatier, 118 route de Narbonne, 31062 Toulouse Cedex, France; 2 Institut Claudius Regaud, 20–24 rue du Pont St Pierre, 31052 Toulouse Cedex, France

**Keywords:** trastuzumab, ovarian cancer, experimental animal models, molecular, mechanisms

## Abstract

**Background:**

The incidence of ovarian cancer has been increasing worldwide and it is currently the leading cause of death from gynaecological malignancy. Unlike breast cancer, the prognostic role of the human epidermal growth factor receptor-2 (HER-2) in ovarian carcinoma remains controversial.

**Methods:**

The aim of this preclinical study was to further characterise the biological, molecular and cellular effects of trastuzumab (Herceptin) using NIH-OVCAR-3 and derived cell lines both *in vitro* and *in vivo*.

**Results:**

*In vitro* assessments have shown that trastuzumab treatment inhibited total and phosphorylated HER-2. This was associated with inhibition of the phosphorylated form of phosphatase and tensin homologue (PTEN), mitogen-activated protein kinase and AKT, and the total level of p27^kip^. Inhibition of PTEN is associated with phosphorylated MEK1/2 upregulation, suggesting a specific inhibition of the protein phosphatase function of PTEN. Moreover, trastuzumab induced the upregulation of RhoB. These molecular modifications promote inhibition of cell migration and potentially restoration of tumour cell contact inhibition. RhoB induction in NIH-OVCAR-3 control cell lines mimics the molecular and cellular trastuzumab long-time exposition effects. RhoB inhibition in NIH-OVCAR-3 long-time exposed to trastuzumab cell line reverses the cellular and molecular effects observed in this model. *In vivo* examinations have shown that these changes are also associated with the restoration of structural, morphological and normal functions of the peritoneum of an ovarian carcinoma mouse model.

**Conclusion:**

These results provide an indication of the mechanisms underlying the anti-tumour activity of trastuzumab that strongly implicate RhoB in an ovarian carcinoma model that does not show HER-2 amplification or overexpression. These findings highlight that trastuzumab effects involve a possible cross-talk between RhoB and PTEN in the early stages of tumour re-growth in a model of micrometastatic ovarian cancer.

The incidence of ovarian cancer has been increasing in the recent decades and it is currently the leading cause of death from gynaecological malignancy (Ozols, 2002). Initially, ovarian cancer is relatively asymptomatic and therefore >50% of patients have advanced or metastatic disease associated with ascites and peritoneal dissemination at the time of diagnosis (Ozols, 2002). Although progress in the routine use of anti-cancer agents, such as platinum analogues and paclitaxel, has improved therapeutic response in advanced ovarian cancer, long-term prognosis remains unsatisfactory (McGuire *et al*, 1996). Peritoneal dissemination of disease is the most common form of progression and recurrence. Both the size of disseminated tumours and the amount of ascitic fluid are known to be inversely correlated with prognosis, and as in any tumour, dissemination is crucial for prognosis (Ozols, 1997).

In retrospective immunohistochemistry analyses, 25% of primary ovarian carcinomas express the human epidermal growth factor receptor-2 (HER-2)-encoded receptor. However, unlike breast cancer, it is controversial to what extent HER-2 amplification and protein overexpression correlate with prognosis (Fan *et al*, 1994; Fajac *et al*, 1995), although it has been reported that HER-2 expression is more frequent in ovarian carcinomas relapsing after chemotherapy (Meden *et al*, 1998). The HER-2 (ERBB2/neu) proto-oncogene encodes for a 185-kDa cell-surface glycoprotein, a transmembrane tyrosine kinase receptor protein that belongs to the epidermal growth factor receptor (EGFR) family (HER-1, HER-2, HER-3, HER-4) (Slamon *et al*, 1989; Baselga and Albanell, 2001; Ross *et al*, 2003). The HER family participates primarily in the transduction of proliferation signal pathways from various ligands, and this process involves dimer formation between different HER receptors. Trastuzumab (Herceptin) is the first anti-HER-2 antibody to be used for the treatment of breast cancer expressing high levels of HER-2 (Press *et al*, 2002; Ross *et al*, 2003) and in their adjuvant setting. In metastatic breast cancer with HER-2 amplification, the addition of trastuzumab to standard chemotherapy yields significantly higher response rates and prolonged survival (Baselga *et al*, 1998; Pegram and Slamon, 1999). *In vitro* and *in vivo* studies have suggested that trastuzumab downregulates the HER-2 receptor and consequently inhibits downstream pathways involved in cell proliferation, survival (Klapper *et al*, 2000; Delord *et al*, 2005) and metastasis (Klapper *et al*, 2000). In breast cancer, trastuzumab dramatically enhances the chemosensitivity of tumour cells. However, the mechanism of action of trastuzumab is still not fully understood, more particularly, trastuzumab potential interactions with the early stages of dissemination of the metastasic process in breast and other cancers.

We have previously reported that trastuzumab as a single agent can cure mice in a micrometastasis model of ovarian carcinoma that do not overexpress HER-2 if started soon after induction chemotherapy (Delord *et al*, 2005). In the same study, we showed that the effects of trastuzumab *in vivo* are associated *in vitro* with a slight inhibition of the proliferative mitogen-activated protein kinase (MAPK) signal transduction pathway and a stronger inhibition of AKT phosphorylation.

The aim of this study was to further characterise the biological, molecular and cellular effects of trastuzumab in human ovarian carcinoma cell models without HER-2 gene amplification or protein overexpression.

## Materials and methods

### Animals and agents

Female Swiss athymic nude mice, 4–5-weeks-old (Charles River Laboratories, L’Arbresele, France) were housed in filtre-capped cages and kept in a sterile facility, which was maintained in accordance with the standards of the Federation of European Laboratory Animal Science Associations. The study was initiated after 2 weeks quarantine. Trastuzumab (Herceptin, TZ) was obtained from F. Hoffmann-La Roche Ltd (Basel, Switzerland).

### Antibodies

The following antibodies were used: anti-Phospho-ErbB2/HER-2 (Tyr1248) provided by Upstate Ab (Euromedex, Mundolsheim, France); anti-total HER-2 (c-erbB-2/HER-2/*neu* Ab-12, clone CB11) and anti-tubulin-β were from NeoMarkers Ab (Interchim, Montluçon, France); anti-active MAPK pAb, rabbit (pTEpY) was from Promega (Charbonnières-les-bains, France); the phospho-AKT antibody (CR 473 Lot-6) and the total AKT antibody (Lot-6) were from Ozyme (Saint-Quentin-en-Yvelines, France); the anti-ERK (c-16) and anti-RhoB rabbit were from Santa Cruz Biotechnology (Tebu-Bio SA, Le Perray en Yvelines, France); the anti-p27^Kip^ was from Dakocytomation (Trappes, France); the anti-total and phosphorylated form of phosphatase and tensin homologue (PTEN) were from Cell Signaling (Ozyme); the anti-phospho-MEK1/2 (Ser218/Ser222) was from Zymed Laboratories (Invitrogen, Cergy Pontoise, France) and the peroxidase-conjugated secondary mouse or rabbit antibodies were from Bio-Rad (Marnes la Coquette, France).

### OVCAR-3 animal model

The Claudius Regaud Institute animal ethics committee approval was obtained for the use of the animal model and the study protocols. The OVCAR-3 tumour model has been described previously (Ortaldo *et al*, 1986; Guichard *et al*, 2001). Briefly, a xenograft in nude mice was obtained after intraperitoneal (i.p.) implantation of 4.5 × 10^7^ cells of the NIH-OVCAR-3 tumour cell line (American Type Culture Collection, ATCC-LGC Promochem Sarl, Molsheim France, HTB-161) and maintained *in vivo* in the laboratory. The i.p. xenograft was passaged as follows: the peritoneal cavity of mice with ascites was irrigated with sodium chloride solution (0.9%), and the wash was combined with ascites. The cells were washed twice in phosphate-buffered saline (PBS). The pellet was resuspended and diluted 1 : 3 in saline solution. Each mouse received 1 ml of this cell suspension i.p., which represented 10–12 × 10^6^ cells. Mice with i.p. tumour xenograft were inspected daily for assessment of overall clinical conditions and assessment of food and water intake. When the tumour reached a macroscopic stage of ascites, mice were inspected twice a day and killed before occurrence of poor health conditions.

### Treatment and microscopic examination

The treated group of nude mice bearing OVCAR-3 macroscopic ascites received 1 ml of trastuzumab administered i.p. at a dose of 150 *μ*g ml^−1^ per day for 24, 48 and 72 h. Naive nude mice were i.p. injected once daily, for 3 days with 1 ml of either RPMI-1640 or the 24-h serum-free cleared supernatant collected from OVCAR-3 cells in culture. Mice were killed and the peritoneum removed, formalin fixed and paraffin embedded. Thick sections of 5 *μ*m from each block were stained with haemalum–eosin and constituted the basis of microscopic examination and photographed.

### Peritoneal permeability test

Peritoneal permeability was assessed using an innovative, simple and convenient technique derived from the method described by Das *et al* (2004). It consists of i.p. injection of 370 MBq (10 mCi) 99^m^ technetium radiolabel diethylene triamine penta-acetic acid (99^m^Tc-DTPA) in nude mice bearing OVCAR-3 macroscopic ascites (treated with or without trastuzumab as described above).

Peritoneal permeability was assessed according to the 99^m^Tc-DTPA elimination from the peritoneal cavity evaluated from the radioactivity distribution in the abdominal region of the mice *vs* its bladder appearance. A dynamic acquisition of planar images was performed using a gamma camera (GEHC – crystal thickness of 5/8″) equipped with low-energy high-resolution collimators. The acquisition setup was as follows.

- Mice were anaesthetised by an i.p. injection of ketamin plus xylazin solution at 100 and 5 mg kg^−1^, respectively.
- They were then placed in a prone position directly on the safety cover of the detector and eight images of equal duration were acquired for a total duration time of 32 min (4 min per frame).
- The matrix size was 256 × 256 with an acquisition zoom of 1.8 corresponding to a pixel size of 12 276 mm.
- Image analysis was performed on a Xeleris workstation (GEHC BUC, Sur Yvette, France; version 2.728 GEHC – Waukesha).

The variation of radioactivity displayed by different organs (such as the abdomen, left and right kidney, bladder) was recorded using elliptical regions of interest (ROIs) surrounding each organs on a summation image (obtained by addition of the eight images of the data set). The ROIs were automatically duplicated on each frame of the data set. Time–activity curves were then plotted for all ROIs and corresponding data were extracted to an EXCEL spreadsheet. Results are expressed as mean±s.e.m. of at least two independent experiments with three mice per group.

### Cell culture

NIH-OVCAR-3 (American Type Culture Collection, ATCC-LGC Promochem Sarl; HTB-161) cells without HER-2 protein overexpression or gene amplification (NIH-OVCAR-3 wild type: OVCAR-3WT) were cultured routinely in RPMI-1640 containing 10% heat-decomplemented serum, supplemented with 20 ng ml^−1^ epidermal growth factor (EGF) (Hoffmann-La Roche Ltd., Meylan, France), 10 *μ*g ml^−1^ insulin (Roche) and 2 mM L-glutamine (Cambrex Biosciences, Emerainville, France) at 37°C and 5% CO_2_. However, oestradiol interferes with the effects of trastuzumab on HER-2 signalling and phenol red acts as an oestradiol-like factor (Treeck *et al*, 2003); therefore, before any *in vitro* study, OVCAR-3 cells were cultured for a week in an otherwise identical medium lacking phenol red.

### OVCAR-3-sublines development

NIH-OVCAR-3 ascites cells (OVCAR-3A) were generated from the mice model of peritoneal ovarian carcinoma as follows. The peritoneal cavity of mice with ascites was irrigated under sterile conditions with sodium chloride solution (0.9%), and the wash was combined with ascites. The cells were washed twice in PBS; the pellet was resuspended in phenol red-free RPMI-1640 medium (as described above) and cultured at 37°C and 5% CO_2_. On the basis of these cells and after 1-month culture, a new model was prepared, NIH-OVCAR-3 ascites cells (OVCAR-3A). These cells were then used to generate a third cell model long-term trastuzumab-exposed cells (OVCAR-3LTE). Briefly, OVCAR-3A cells were cultured in phenol red-free RPMI-1640 medium containing 10% heat-decomplemented serum and treated three times a week with trastuzumab 150 *μ*g ml^−1^ during 6 months.

### RhoB upregulation, Adeno-RhoB: adenoviral constructs and transduction protocol

Replication-defective (E1,E3) Ad vectors expressing RhoB under the transcriptional control of the CMV promoter were constructed using the AdEasy System (Qbiogen, F, Illkirch, France). Initially, a BglII-BamH1 fragment containing RhoB and obtained from digestion of the pGR5-2-RhoB plasmid already described was subcloned into BamHI-digested pAdenoVATOR-CMV5(CuO)-ires GFP transfer vector (Qbiogen, F) to obtain the shuttle vector pAdRhoB. This vector allows transgene expression driven by the cumate-inducible CMV5(CuO) promoter. An internal ribosome entry-site sequence ensures co-expression of GFP. The replication-defective vector AdpRhoB and the control empty vector AdeGFP (encoding enhanced green fluorescent protein) were produced as described previously (Malecaze *et al*, 2006). Viral titres were determined by optical absorbance at 260 nm (1 OD=1 × 1012 physical particle per ml). A total of 140 × 10^3^ cells were plated on a 35-mm dish 24 h before transduction. On day 0, cells were transduced with viral vectors at an MOI of 10/1. Cells were then amplified and used for experiments of western blot analysis of RhoB, PTEN, MEK1-2 expression and/or activity, immunofluorescence assay for actin network organisation and density evaluation and migration assay as described next in this study.

### RhoB inhibition ShRNA RhoB: lentiviral vector

LVTHM is a lentiviral vector (LV) encoding the shRNA RhoB1 (5′-CGCGTCCCCGGCATTCTCTAAAGCTATGTTCAAGAGACATAGCTTTAGAGAATGCCTTTTTGGAAAT-3′) or RhoB2 (5′-CGCGTCCCCTTGATATCCCTTGTCTGTAATTCAAGAGATTACAGACAAGGGATATCAATTTTTGAAA-3′) under the control of the H1 promoter. 293T cells were kindly provided by Genethon (Evry, France). Generation of 293T-LVTHM-shB2 and preparation of high-titre LV pseudotyped with the VSV-G protein have been described previously. Viruses containing supernatants were prepared as described elsewhere (Couderc *et al*, 2008). In all, 1 × 10^6^ cells were plated on 100-mm dishes 24 h before transduction with viral vectors at an MOI of 10 : 1. Cells were then used for experiments of western blot analysis of RhoB, PTEN, MEK1-2 expression and/or activity, immunofluorescence assay for actin network organisation and density evaluation and migration assay as described next in this study.

### Cell growth determination

OVCAR-3WT, OVCAR-3A or OVCAR-3LTE cells were seeded in RPMI-1640 phenol red-free medium containing 10 or 2.5% heat-decomplemented serum at 2000 and 4000 cells per well for OVCAR-3WT and OVCAR-3A, respectively, and 2000 and 3000 cells per well for OVCAR-3LTE in a 96-well plate in sextuplet on day 0. On day 1 and every day, cells were treated with either vehicle or trastuzumab in a concentration range of 0–600 *μ*g ml^−1^. The number of cells was evaluated on day 0 (18 h after plating) and every 24 h as indicated by a sulforhodamine B test described elsewhere (Skehan *et al*, 1990). Briefly, sulforhodamine B is a colorimetric assay determining the protein sample content directly correlated with the number of cells.

### Phosphorylated quantification

OVCAR-3WT, OVCAR-3A or OVCAR-3LTE cells were plated at 1 × 10^6^ cells per 100-mm culture dishes in RPMI-1640 phenol red-free medium supplemented with 10% heat-decomplemented serum and 2 mM L-glutamine in 100-mm culture dishes. Cells were serum starved for 24 h and then treated with either trastuzumab (150 *μ*g ml^−1^) or vehicle for 24 h. When indicated, EGF stimulation (20 ng ml^−1^) was performed during the last 15 min of treatment. Concentration of phosphorylated EGFR was measured using an active EGFR enzyme immunoassay (EIA) Kit (Takara Biomedicals, Tokyo, Japan) following the supplier's instructions and expressed as the amount of phosphorylated EGFR (fmol ml^−1^).

### Fluorescence

Cells were seeded on glass coverslips into 6-well plates (Nunc Inc., Naperville, IL, USA) at a density of 8 × 10^4^ or 1 × 10^5^ cells per well in RPMI-1640 phenol red-free medium supplemented with 10% heat-decomplemented serum and 2 mM L-glutamine. After 48 h cells were serum starved or not for 48 h. Cells were then fixed in 3% paraformaldehyde and permeabilised into 0.1% Triton X-100 in PBS. Actin fibres were detected by incubation with tetramethylrhodamine isothiocyanate-labelled phalloidin (Molecular Probes, Invitrogen). Cells were viewed on a Zeiss Axiophot microscope (Zeiss, Sartrouville, France), and photographed using a Princeton camera (Princeton, Sartrouville, France).

### Focus-formation assay

OVCAR-3WT, OVCAR-3A or OVCAR-3LTE cells were seeded at 1 × 10^6^ cells in 60-mm culture dishes in RPMI-1640 phenol red-free medium supplemented with 10% heat-decomplemented serum and 2 mM L-glutamine. For OVCAR-3WT, OVCAR-3A studies were also performed in RPMI-1640 containing 10% heat-decomplemented serum, supplemented with 20 ng ml^−1^ EGF, 10 *μ*g ml^−1^ insulin and 2 mM L-glutamine. Cell foci were scored 15 days after cell confluence and photographed.

### Plating efficiency determination

OVCAR-3WT, OVCAR-3A or OVCAR-3LTE cells were seeded at 300 cells per 60-mm culture dishes in RPMI-1640 phenol red-free medium supplemented with 10% heat-decomplemented serum and 2 mM L-glutamine. For OVCAR-3WT, OVCAR-3A experiments were also performed in RPMI-1640 containing 10% heat-decomplemented serum, supplemented with 20 ng ml^−1^ EGF, 10 *μ*g ml^−1^ insulin and 2 mM L-glutamine. Cell plating efficiency was scored 10 or 15 days later, respectively, by fixing with ethanol–formol–acetic acid (75%/20%/5%) and staining with crystal violet.

### Anchorage-independent growth assays

Cells were seeded at 1000 cells per well in 12-well plates in duplicate in 0.3% agar over a 0.6% agar layer. Cells were fed twice weekly until colonies grew to a suitable size for observation (∼12 days). Colonies were photographed after 4 h of incubation with 1 mg ml^−1^ diphenyltetrazolium bromide in RPMI at 37°C. The growth of OVCAR-3WT and OVCAR-3A colonies was compared with that of OVCAR-3LTE colonies.

### Migration assay

Cells were plated at 1 × 10^6^ per 60-mm culture dishes in RPMI-1640 phenol red-free medium supplemented with 10% heat-decomplemented serum and 2 mM L-glutamine for OVCAR-3LTE and with 20 ng ml^−1^ EGF and 10 *μ*g ml^−1^ insulin for OVCAR-3WT and OVCAR-3A, respectively. When cells were confluent, ‘scratch’ wounds were created by scraping confluent cell monolayers with a sterile pipette tip to make a gap. The scraped down cells were washed by serum-free medium and re-fed with the appropriate medium. Migration was scored and photographs taken on the ‘scratch’ wound day and every 12 h.

### Western blot analysis

On day 1, 1.5 × 10^6^ OVCAR-3WT, OVCAR-3A or OVCAR-3LTE cells were plated in phenol red-free RPMI-1640 medium containing 10% heat-decomplemented serum supplemented with 2 mM L-glutamine and 20 ng ml^−1^ EGF, 10 *μ*g ml^−1^ insulin for OVCAR-3WT, OVCAR-3A in 100-mm culture dishes. After 48 h, cells were serum starved and treated with either vehicle or trastuzumab 150 *μ*g ml^−1^ for 24–72 h. Before the end of the experiment and when necessary, cells were treated with 20 ng ml^−1^ EGF for the last 15 min of the experiment. The cells were harvested and lysed in lysis buffer (Tris 50 mM pH 8, NaCl 150 mM, 0.1% NP40, 5 mM MgCl_2_, 50 mM NaF, 2 mM PMSF, 10 mM DTT, 2 mM orthovanadate, 5 mg ml^−1^ sodium dexoxycholate, 6.4 mg ml^−1^ phosphatase substrate; Sigma, Lyon, France; 104). For HER-2, HER-1, MAPK, AKT, p27^Kip^, PTEN, MEK, RhoB or β-tubulin analysis, 70 *μ*g of the cleared lysates were separated on a 7.5 or 12.5% sodium dodecyl sulphate polyacrylamide gel blotted to PVDF membranes (Amersham Pharmacia Biotech, Orsay, France), and incubated with specific antibodies. Detection was performed using peroxidase-conjugated secondary antibodies (Bio-Rad) and an enhanced chemiluminescence detection kit (Amersham Pharmacia Biotech). Blots were scanned and analysed and quantified with Molecular Dynamics (Saclay, France) densitometer and ImageQuant software (GE Healthcare, Saclay, France).

### Statistical analysis

All results are expressed as mean±s.e.m. Results were analysed using Student's *t*-tests, and a *P*-value <0.05 was accepted as statistically significant.

## Results

### *In vivo*

#### Pathological effects of trastuzumab

**Figure 1:**
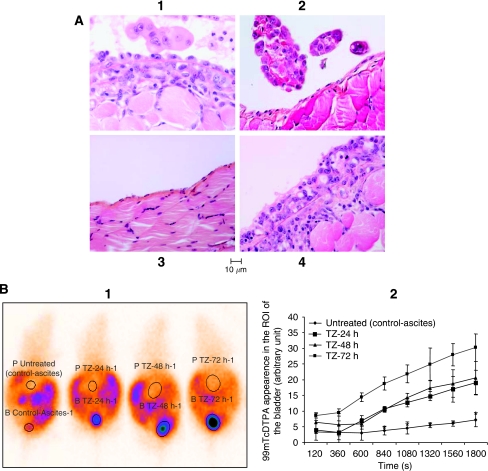
(**A**) Pathological pattern of the peritoneum 20 days after OVCAR-3WT implantation. Nude mice bearing OVCAR-3 macroscopic ascites were treated with vehicle (**A1**) or trastuzumab at a dose of 150 *μ*g ml per day (**A2**) administered by i.p. route for 72 h. Naive nude mice were i.p. injected once a day for 3 days with 1 ml of either RPMI-1640 (**A3** control group) or the 24 h serum-free cleared supernatant collected from OVCAR-3 cells in culture (**A4**). Mice were killed and the peritonea were collected and formalin fixed. Microscopic examination was performed and pictures were taken. (**B**) Concentrations of 99^m^Tc-DTPA over time in the bladder: peritoneal permeability. 99^m^Tc-DTPA appeared in the bladder more intensively in treated animals compared with the untreated group. Arbitrary units express the 99^m^Tc-DTPA intensity detection in the region of interest (ROI) of the bladder (shortened by ‘B’ and by ‘P’ for the peritoneum:). ^*^P<0.05. Student's *t*-test compared untreated group (control-ascites) with trastuzumab (TZ) treatment groups.

The pathological pattern of the peritoneum was evaluated 20 days after OVCAR-3WT implantation driving to macroscopic peritoneal ascites (Figure 1A). We compared the naive nude mice group injected with RPMI-1640 (Figure 1A3), which is the control group of Figure 1A, with the untreated mice group (Figure 1A1) and trastuzumab-treated animals (Figure 1A2). Mice were killed and the peritonea were dissected to assess the pathological morphology of the peritoneum. Compared with the control group (naive nude mice injected with RPMI-1640: Figure 1A3), untreated mice displayed inflammatory modifications of the peritoneum (Figure 1A1). These lesions consisted of mesothelial hyperplasia on the surface of plump and non-cohesive mesothelial cells and in the underlying layers of vascular and fibroblastic granulation tissue. Tumour cell clusters infiltrating the peritoneum were focally seen. By contrast, trastuzumab treatment dramatically decreased the number and size of tumour cell clusters infiltrating the peritoneum (Figure 1A2). It also induced restoration of a normal peritoneal structure with a continuous surface layer of flattened cells similar to the light microscopic characteristics of the normal peritoneum in the control group (Figure 1A3), and decreased the vascular and inflammatory components.

To clarify whether these modifications were due to a direct trastuzumab effect on the peritoneum or a biological effect of trastuzumab on OVCAR-3 cells, naive nude mice were i.p. injected once a day, for 3 days with 1 ml of either RPMI-1640 (Figure 1A3) or a 24 h serum-free cleared supernatant collected from OVCAR-3WT cultured cells (Figure 1A4). We observed that the i.p. injection of the cleared supernatant collected from OVCAR-3WT cultured cells (Figure 1A4) induced the same inflammatory changes of the peritoneum (that is, mesothelial hyperplasia, plump and non-cohesive mesothelial cells and the underlying granulation tissue) observed in the untreated groups of the peritoneal ovarian carcinoma (Figure 1A1) and not observed in the control group (RPMI-1640-injected mice), thus showing that trastuzumab action is related to a cellular effect on OVCAR-3 cells and not on the mice peritoneum.

#### Effects of trastuzumab on peritoneal permeability

Peritoneal permeability was assessed according to the 99^m^Tc-DTPA kinetics of elimination from the peritoneal cavity (P) and its appearance in the bladder (Figure 1B and C). Elimination of 99^m^Tc-DTPA from the peritoneal cavity increased in mice treated with trastuzumab compared with untreated mice, the control group of the Figure 1B and named untreated control-ascites. Higher concentrations of 99^m^Tc-DTPA appeared in the bladders (B) of treated animals compared with control mice (*P*<0.05; Student's *t*-test). These data are the consequence of peritoneal dialysis restoration in treated animals in relation to the restoration of the peritoneum structure we have described previously. Results are expressed as mean±s.e.m. of at least two independent experiments with three mice per group.

### *In vitro*

*In vitro* experiments were performed with OVCAR-3WT at an early passage, OVCAR-3WT cultured 6 months, OVCAR-3A at a early passage and OVCAR-3A cultured 6 months, because we sought to not only address the contribution of artefacts due to long-term culture to our observation but also to justify that the observed effects are caused by long-time treatment with trastuzumab and not due to the cumulative acquired mutations that could be induced by a long-term culture. Our *in vitro* data did not show any significant difference between OVCAR-3WT, OVCAR-3A and the culture passage (data not shown), leading us to present only the result observed with the 6-month cultured OVCAR-3WT, OVCAR-3A to be homogenous with the long-time exposure to trastuzumab cell line, the OVCAR-3LTE subclone.

#### Cell line proliferation and effects of trastuzumab

**Figure 2:**
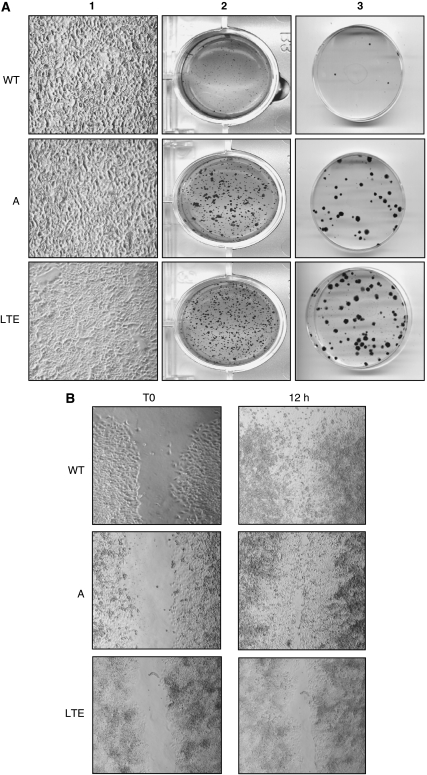
During the focus-formation assay, OVCAR-3A and WT cells grew at a high density and formed numerous foci (**A1**). Anchorage-independent growth assays showing the development of clones in OVCAR-3A and LTE cells (**A2**). Plating efficiency. (**A3**) Migration assay, when ‘scratch’ wounds were created by scraping with a sterile pipette tip to make a gap. Only OVCAR-3LTE cells were not capable of settling the created ‘scratch’ wounds (**B**).

Regardless of the heat-decomplemented serum concentration (10 or 2.5%), OVCAR-3LTE cells proliferated more quickly than did the two other cell lines, which have similar growth characteristics. At 2.5% heat-decomplemented serum, OVCAR-3WT, A and LTE cell lines have a lag time of 24 h and a doubling time of 46, 45 and 27.5 h, respectively (Figure 2A).

When the three OVCAR-3 cell line models were maintained in RPMI-1640 medium containing 10% heat-decomplemented serum, trastuzumab had no notable anti-proliferative effect (data not shown). However, when OVCAR-3WT and A (data not shown for OVCAR-3A because growth profiles were identical to those of OVCAR-3WT) cells were cultured in a lower heat-decomplemented serum concentration (2.5%) and in phenol red-free RPMI-1640 medium, trastuzumab moderately inhibited cell proliferation in a dose-dependent manner. This inhibition is statistically significant when comparing untreated cells with trastuzumab-treated cells at 75, 150 or 300 *μ*g ml^−1^ as previously reported (Delord *et al*, 2005) (Figure 2B). In contrast, OVCAR-3LTE cells were not affected.

#### Effect of trastuzumab on cellular functions

**Figure 3:**
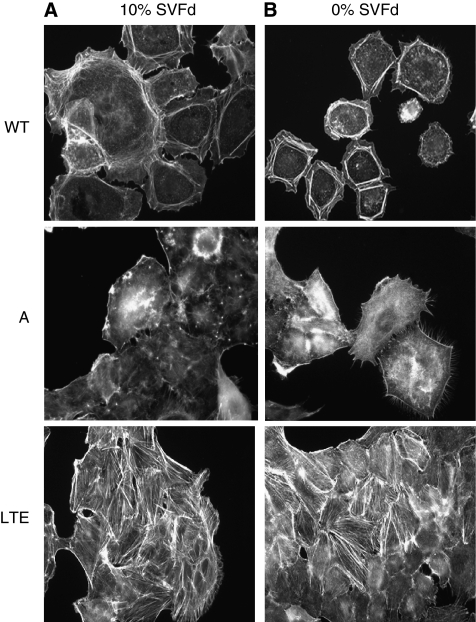
Actin network. OVCAR-3LTE cells display a dense and well-organised actin cytoskeleton network at 10% heat-decomplemented serum or under serum-starvation conditions (**A** and **B**, respectively).

We initially compared OVCAR-3WT, A and LTE cell lines for plating efficiency and anchorage-independent growth assays and then for focus formation and migration capacity. For plating efficiency (Figure 3A3) and anchorage-independent growth assays (Figure 3A2), using the soft agar culture test, OVCAR-3A or LTE cells developed clones after plating under drastic conditions (300 cells in 60-mm dishes) and clones in soft agar regardless of the cultured conditions (such as a medium with or without EGF and insulin at 20 ng ml^−1^ and 10 *μ*g ml^−1^ for OVCAR-3WT or A, respectively), OVCAR-3WT cell lines did not have these potentialities (Figure 3A2 and A3).

For focus-formation assay, regardless of culture conditions (medium with or without EGF and insulin for OVACR-3WT and A), OVCAR-3LTE cells grew only in a monolayer, whereas OVCAR-3A and WT cells grew at a higher density and formed numerous foci (Figure 3A1).

Ovarian cancer cells are described in the literature for having a strong metastatic potential. Therefore, we assessed the migration properties and showed that only OVCAR-3LTE cells were unable to settle on created ‘scratch’ wounds, demonstrating that chronic trastuzumab treatment induced the inhibition of cell-migration properties (Figure 3B).

#### Effect of trastuzumab on actin cytoskeleton organisation of OVCAR-3WT, A and LTE cells

Cellular motility capacity implies a dynamic regulation of the actin cytoskeleton and particularly those of the cortical network. Thus, we determined whether long-time exposure to trastuzumab could induce any actin network remodelling by actin immunostaining using phalloidin toxin coupled with rhodamine fluorochrome.

**Figure 4:**
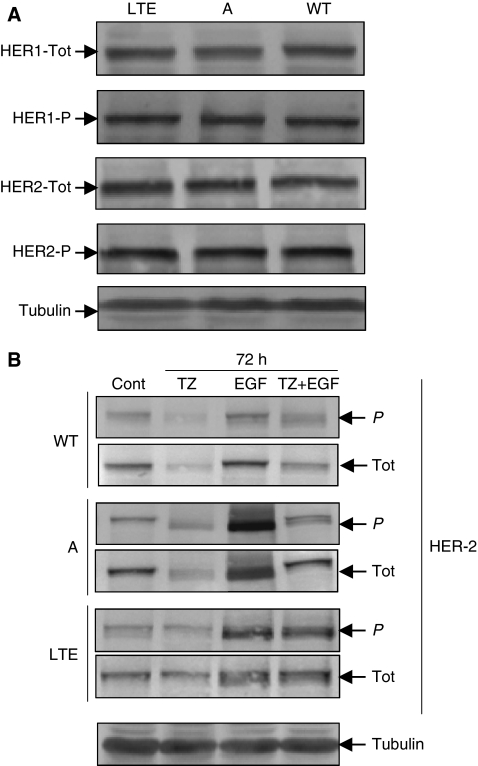
Total and phosphorylated levels of HER-1 and HER-2 expression in serum-starvation condition: no significant differences between cell lines (**A**). After 72 h trastuzumab treatment, phosphorylated and total HER-2 levels were significantly inhibited under basal (0% heat-decomplemented serum) and EGF-induced conditions in OVCAR-3WT and A cells (**B**).

The actin cytoskeleton in OVCAR-3WT and A is similar and different from cytoskeleton in OVCAR-3LTE. As shown in Figure 4, and regardless of culture conditions (medium with or without heat-decomplemented serum), OVCAR-3WT and A cells display only a cortical actin network, which is not observed in OVCAR-3LTE cells. By contrast, only OVCAR-3LTE cells present a well-organised actin cytoskeleton network at 10% heat-decomplemented serum or under serum-starved conditions.

#### Effect of trastuzumab on HER-1 and 2 receptors and downstream target

**Figure 5:**
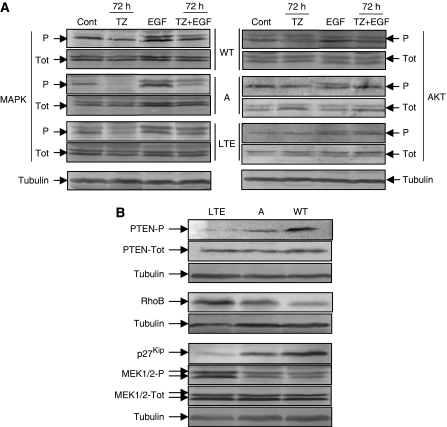
Total and phosphorylated protein levels. Trastuzumab treatment (from 24 to 96 h) inhibited MAPK and AKT EGF-induced-phosphorylation of OVCAR-3WT and A cell lines under serum-starvation conditions. (**A**) Western blot analysis showing in OVCAR-3LTE cells: inhibition of the active form of PTEN and total p27^kip^, upregulation of phosphorylated MEK1/2, and higher RhoB protein level expression (**B**).

Under serum-starved conditions, no significant differences were observed in total and phosphorylated levels of HER-1 expression between the three cell lines (Figure 5A and EIA kit data not shown). Moreover, long-term trastuzumab exposure has no effect on total (Figure 5A) or basal phosphorylation (Figure 5A) levels of HER-2 between the three cell lines.

When treated with trastuzumab for 72 h, we observed that OVCAR-3WT and A cells showed a significant inhibition of both phosphorylated and total HER-2 levels under basal (0% heat-decomplemented serum) and EGF-induced conditions (Figure 5B), showing that trastuzumab binds to the extracellular domain of HER-2 and inhibits EGF ligand-induced phosphorylation. However, in OVCAR-3LTE cells, these effects were not or very weak if observed.

#### Molecular mechanisms involved in trastuzumab action

**Figure 6:**
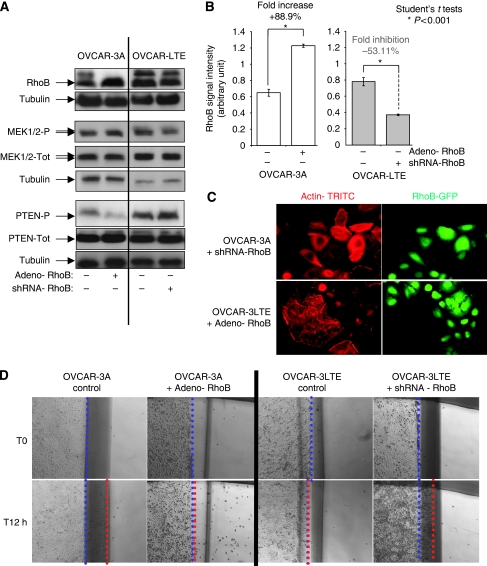
(**A**) Western blot analysis showing that inhibition of RhoB (shRNA-RhoB) in OVCAR-3LTE reverses the downregulation of phospho-PTEN and the upregulation of phospho-MEK1/2. Induction of RhoB (adeno-RhoB) in OVCAR-3A mimics the PTEN and MEK1/2 phosphorylation regulation observed in OVCAR-3LTE (panel A). (**B**) Histogram represents the quantisation of the statistically significant RhoB induction (adeno-RhoB, +88.9%) in OVCAR-3A and RhoB statistically significant downregulation (shRNA-RhoB, −53.11%) (panel B) observed by western blot (panel A). (**C**) Actin network. Inhibition of RhoB (shRNA-RhoB) in OVCAR-3LTE reverses actin cytoskeleton network organisation and density as observed in control cell lines (OVCAR-3A). Induction of RhoB (Adeno-RhoB) in OVCAR-3A cells mimics the actin cytoskeleton network organisation and density observed in OVCAR-3LTE cells (panel C). (**D**) Migration assay, when ‘scratch’ wounds were created by scraping to make an gap, RhoB inhibition (shRNA-RhoB) in OVCAR-3LTE restores migration capacities observed in control cell lines; on the contrary, induction of RhoB (Adeno-RhoB) in control cell lines (OVCAR-3A and OVCAR-3WT-data not shown) inhibits their migration as observed in long-time trastuzumab-exposed cell line OVCAR-3LTE (panel D).

Under serum-starved conditions, trastuzumab treatment (from 24 to 96 h) inhibited MAPK and AKT EGF-induced phosphorylation of OVCAR-3WT and A cell lines (Figure 6A). Under the same conditions, MAPK and AKT phosphorylations were not, or very slight when observed, affected in OVCAR-3LTE cells.

#### Effect of trastuzumab on PTEN pathway and p27^kip^

Using western blot analysis, the status of PTEN and p27^kip^ expression was assessed in the three cell lines. As shown in Figure 6B, the active form of the PTEN protein and total p27^kip^ were strongly inhibited in OVCAR-3LTE cells compared with the two other cell lines, whereas the phosphorylated form of MEK1/2 was upregulated in OVCAR-3LTE cells compared with the two other cell lines (Figure 6B).

#### Effect of the trastuzumab on RhoB

The loss of the migratory capacity and the modification of the actin cytoskeleton distribution/structure/organisation of the OVCAR-3LTE cell could be associated with the modification of RhoB expression. Using western blot, we evaluated RhoB expression in the three cell lines (Figure 5B) and showed that OVCAR-3LTE cells have a higher RhoB protein expression level than do OVCAR-3WT and OVCAR-3A cells. Moreover, trastuzumab treatment (150 mg for 72 h) induced upregulation of RhoB in all cell lines (data not shown) and was associated with the migratory inhibition observed in OVCAR-3LTE cells. Furthermore, these data highlight a striking inverse correlation between RhoB expression and PTEN phosphorylation or p27 expression.

#### RhoB implication in trastuzumab effects

To decipher the RhoB implication in trastuzumab effects, we chose to study its expression regulation in control cells (OVACR-3A and WT) by its upexpression (Adeno-RhoB) and in the long-time exposed derived cells its inhibition (OVCAR-3LTE, shRNA RhoB). As the observed regulation and the collected results were fully similar between OVCAR-3A and WT in the studied properties, we chose to show in the figures only those of the OVCAR-3A cell line we have used to generate the cell model long-term trastuzumab-exposed cells (OVCAR-3LTE). We also studied their actin cytoskeleton organisation, migration capacities and their regulation of PTEN and MEK1-2 activity. We showed that RhoB induction (Figure 6A and B, +88.9% significant induction: *P*<0.001 – Student's *t*-tests) in OVCAR-3A (open bars) mimic the actin cytoskeleton network organisation and density (Figure 6C), inhibit their migration capacities (Figure 6D) and PTEN and MEK1/2 phosphorylation regulation observed in OVCAR-3LTE (Figure 5B). On the contrary, the inhibition (Figure 6A and B, −53.11% significant inhibition: *P*<0.001 – Student's *t*-tests) of RhoB in OVCAR-3LTE (grey bars) drive us to observe the OVCAR-3A properties in actin cytoskeleton organisation and density (Figure 6C), migration capacities (Figure 6D) and their regulation of PTEN and MEK1-2 (Figure 6A) activity we previously described (Figure 5B).

## Discussion

Trastuzumab binds specifically to the extracellular domain of HER-2 and can induce downregulation and inactivation of the receptor through several mechanisms, including inhibition of the cell cycle and the survival pathways. Inactivation, in turn, results in poorly understood anti-tumoural responses, although trastuzumab prolongs survival in breast cancer patients when administered in association with chemotherapy. Furthermore, many patients have primary resistance to trastuzumab for unknown reasons (Nagata *et al*, 2004). However, Nagata *et al* (2004) have provided data that clarify the anti-tumoural mechanism of trastuzumab and the mechanism underlying resistance. They showed that, on binding to the HER-2 receptor, trastuzumab stabilises and activates the PTEN tumour suppressor and consequently downregulates the PI3K-AKT signalling pathway. We have also previously reported that trastuzumab can cure mice in a model of ovarian carcinoma when administered in the early stage of relapse (Delord *et al*, 2005). These *in vivo* and *in vitro* trastuzumab effects were associated with a slight inhibition of the proliferative signal transduction pathway (MAPK) and a stronger inhibition of the PI3K/AKT pathway (Delord *et al*, 2005).

In this study, we have shown that trastuzumab can restore the peritoneal structure and normal permeability previously affected by cancer cells. Trastuzumab modifies peritoneal clearance and the cancer-peritoneal cross-talk in animals with macroscopic carcinomatosis. Trastuzumab was shown to modify cancer cell functions and biology through the regulation of several molecular targets, such as PTEN, MEK1-2 and RhoB. These results suggest that the anti-invasive function of trastuzumab leading to cure mice in a micrometastasis model of ovarian cancer involves a possible cross-talk between RhoB and the PTEN pathway.

All normal serous membranes, such as the peritoneum, consist of a single layer of flat mesothelial cells resting on a basement membrane, with a submesothelial layer of connective tissue involved in fluid transport. In the murine model of ovarian carcinoma, substantial alterations to the peritoneum were observed both at the macroscopic and submacroscopic levels. Such alterations may include thickening of the surface membrane with malignant ascites formation and infiltration. At the microscopic level, there may be multilayering of the surface epithelium (hyperplasia) and an inflammatory infiltrate composed of different leukocyte populations. The administration of serum-free supernatant collected from OVCAR-3WT-cultured cells induced the same inflammatory and structural changes of the peritoneum as carcinomatosis. Trastuzumab reversed these modifications and induced the restoration of peritoneal dialysis. These results suggest that effect of trastuzumab on ovarian carcinoma cells may result from inhibition of one or more secreted molecules or pathways involved in the processes of anchorage, proliferation and migration of ovarian carcinoma. The identification of these molecules and pathways is currently under investigation.

We have previously shown that proliferation of OVCAR-3LTE cells is higher compared with control cell lines (Delord *et al*, 2005), suggesting once again that the inhibition of the cellular proliferation induced by trastuzumab does not fully explain its mechanism of action. The inhibition of proliferation is certainly a significant mode of action of trastuzumab in cellular models overexpressing the HER-2 receptor (Longva *et al*, 2005), but OVCAR-3 cells and the derived subclones used in this study do not amplify or overexpress HER-2. Our results suggest that trastuzumab is also effective in models that do not overexpress HER-2 when used in minimal disease, during the early stages of repopulation and invasion.

To determine whether trastuzumab induces modifications of cellular functions, comparisons were first made between OVCAR-3WT, A and LTE cell lines in terms of plating efficiency and anchorage-independent growth assays. Only OVCAR-3A and LTE cells were able to develop clones after plating under drastic conditions and in soft agar. This indicates that OVCAR-3WT *in vivo* passage contributed to select clones with these cellular properties, which cannot be correlated with trastuzumab long-time exposure, but only to *in vivo* passage. Loss-of-contact inhibition properties constitute an important characteristic of transformed cells. This was assessed by focus-formation assays and migration capacity. Restoration of cellular contact inhibition and inhibition of cellular migration capacity was observed in OVCAR-3LTE. These results strongly suggest that the trastuzumab effect could modify two major functions of transformed cells: contact inhibition and invasion.

On the basis of these results, we hypothesised that in mice, trastuzumab treatment could inhibit the early stages of tumour growth, migration and invasion, which probably are key processes of early tumour re-growth. The cellular migration phenomenon relies on a complex protein network of actin filaments, which extend across the cytoplasm associated with a cortical network and participates in the regulation of the filopodia, lamellipodia or ruffles formation (Hall, 2005). This network is a dynamic multiproteic structure, which reorganises the cell morphology continuously during cell division or in response to different stimuli (Gov and Gopinathan, 2006).

Members of the Rho GTPase family are mediators of diverse cellular functions, such as the regulation of actin cytoskeleton organisation or migration (Hall, 2005). Although Cdc42 promotes filopodia formation, Rac1 promotes formation of lamellipodia and membrane ruffles, RhoA triggers actin stress fibre and focal adhesion formation (Kozma *et al*, 1995; Nobes *et al*, 1998). Consequently, Rho GTPase functions can influence cell–cell and cell–substratum interactions, which in turn can modulate cell movement. From actin network studies, we showed that trastuzumab chronic treatment of OVCAR-3LTE cells induced the formation of a cytoplasmic actin stress fibre network. As described by Nobes *et al* (1998), the cortical network is linked to the function of Rac and Cdc42 proteins (Kozma *et al*, 1995) and is also associated with cellular migration, motility and invasion (Keely *et al*, 1997). These data are in agreement with our results showing a correlation between the absence of this cortical network and the inhibition of the migratory capacity observed in the OVCAR-3LTE cell line.

It is well known that the GTPases of the Rho family such as RhoA, RhoB and RhoC are implicated in the control of actin cytoskeleton rearrangement and thus in cellular migration (Van Aelst and D’Souza-Schorey, 1997; Hall, 2005). We show in this study that RhoB induction in control cell lines (OVCAR-3A and WT) and its inhibition in OVCAR-3LTE is critical in ovarian cell lines we used for migration capacities and certainly for tumour aggressiveness together induced by trastuzumab treatment. Mazières *et al* (2004) demonstrated in a study on human lung cancer tissues, a correlation between RhoB loss of expression and a worse tumour stage. These data suggest that RhoB is implicated in the regulation of malignant transformation and progression. Moreover, Jiang *et al* (2004) identified suppression of RhoB as a mechanism inducing tumour metastasis. In our cell lines, trastuzumab treatment induced an increase in RhoB protein levels, which is associated with the inhibition of cellular migration. To validate the link between trastuzumab, RhoB and these properties controlled by actin cytoskeleton and cortical network organisation and regulation, we performed a series of experiments of RhoB expression variation. We have shown that inhibition of these properties are concomitant to the upregulation of RhoB in control cells lines (OVCAR-3WT and A) and their restorations are associated with RhoB inhibition in the long time exposed to the trastuzumab cell line (OVCAR-3LTE). The data presented in this study together with the available literature demonstrate that trastuzumab action in the repopulation stage involves cytoskeleton modulation and migration properties by the regulation of the Rho GTPase family and more specifically RhoB.

Elsewhere, we have also assessed whether trastuzumab treatment could induce a positive regulation of HER-1, which could be involved in trastuzumab resistance after long exposure (Delord *et al*, 2005). The level of phosphorylated and total HER-1 was measured in the three cell lines and there was no observed difference. Moreover, in OVCAR-3LTE cells, we observed the loss of HER-2 and downstream pathway regulation, indicating that the receptor and pathway of these cells were modified in response to the drug. The long-time exposure to trastuzumab could render the HER-2 receptor of this cell line capable of bypassing trastuzumab inhibition. However, this mechanism has not been fully investigated in this study as it was a not primary objective.

In breast cancer models, many *in vitro* studies have indicated a link between the effects of trastuzumab and PTEN (Nahta *et al*, 2004; Pandolfi, 2004) and p27^kip^. In this study, we showed in OVCAR-3LTE the loss of the phosphorylated form of PTEN (deleted from chromosome 10) and a strong inhibition of the total form of p27^kip^ associated with the upregulation of the phosphorylated form of the protein MEK1/2 and RhoB upregulation. Moreover, these observed regulations highlight an inverse correlation between RhoB expression and PTEN and p27 downregulation, strongly suggesting the RhoB implication in the trastuzumab action and certainly resistance.

Nagata *et al* (2004) reported that the loss of PTEN expression in breast cancer cell lines overexpressing HER-2 confers resistance to trastuzumab. The data from the present ovarian cell line studies show that response to trastuzumab involves the regulation of PTEN and p27^kip^ expression. The inhibition of PTEN activity observed in this study could be partly responsible for the insensitivity to trastuzumab in terms of the proliferation observed in OVCAR-3LTE cells. Indeed, PTEN is a tumour suppressor involved in the regulation of many cellular process, such as regulation of the cell cycle (G1/S), apoptosis, the rearrangement of the cytoskeleton and migration (Wu *et al*, 2003; Raftopoulou *et al*, 2004). Moreover, PTEN is reported to have at least two main molecular functions: (Wu *et al*, 2003) protein phosphatase and lipid phosphatase activity.

The protein phosphatase activity is involved in the inhibition of growth factor-stimulated MAPK signalling. The loss of PTEN protein phosphatase activity induces the expression of the phosphorylated form of MEK1/2 as observed herein in OVCAR-3LTE cells. The protein phosphatase activity is also involved in the reorganisation of the actin cytoskeleton (Attwell *et al*, 2003), the regulation of migration (Raftopoulou *et al*, 2004) and the cellular invasion process (Sanchez *et al*, 2005) by inhibition of focal adhesion kinase. Indeed, PTEN, by its protein phosphatase activity is responsible for the inhibition of cellular migration. In our model, PTEN phosphatase activity is possibly preserved despite a strong decrease in its phosphorylated form. Consequently, migration properties are not affected in the cell line long-time exposed to trastuzumab. As discussed, RhoB is probably involved in the changes observed in migration. Furthermore, and for the fist time for trastuzumab molecular effects, we showed a direct link between PTEN activity and RhoB expression both related to trastuzumab action. We have shown that inhibition of PTEN phosphorylation level is concomitant to the upregulation of RhoB expression in control cells lines (OVCAR-3WT and A), and on the contrary, RhoB inhibition in the long-time exposed to trastuzumab cell line (OVCAR-3LTE) is associated to PTEN phosphorylation increase. This PTEN regulation process, we imply to trastuzumab under the RhoB control, is corroborated by the MEK1/2 phosphorylation level modification that we describe in the experiments of induction/inhibition of RhoB expression we performed. Indeed, a cross-talk effect between RhoB and PTEN could strongly be evoked in the cellular effect of trastuzumab. RhoB expression modulation could be similar to PTEN regulation (Nagata *et al*, 2004), a marker of the biological and molecular effect of trastuzumab in our model.

The second function of PTEN is the inhibition of AKT by lipid phosphatase activity, which has a major role in anti-proliferative and pro-apoptotic mechanisms. Indeed, the PTEN lipid phosphatase activity contributes to the regulation of the cellular cycle by p27^kip^ induction. Trastuzumab resulted in p27^kip^ protein levels in OVCAR-3LTE cells. p27^kip^ is a cyclin-dependent kinase inhibitor involved in the inhibition of cell-cycle progression in the G1/S phase. The p27^kip^ inhibition seen in OVCAR-3LTE cells could be involved in the stimulation of proliferation that was observed. Nevertheless, the increase in proliferative capacities has no affect on the invasive properties in our model.

The data presented in this study suggest that trastuzumab properties are complex, but also highlight a significant effect of this agent in the early stages of tumour invasion as a consequence of PTEN and RhoB regulation. Further experiments will be developed with complementary ovarian carcinoma cell lines overexpressing HER-2 receptor to decipher the role of RhoB/PTEN cross-talk and/or expression *vs* the HER-2 expression level to increase our knowledge in ovarian carcinomas treatment.
